# Treatment-Resistant Depression Revisited: A Glimmer of Hope

**DOI:** 10.3390/jpm11020155

**Published:** 2021-02-23

**Authors:** Angelos Halaris, Emilie Sohl, Elizabeth A. Whitham

**Affiliations:** Department of Psychiatry, Loyola University Stritch School of Medicine, Maywood, IL 60153, USA; esohl@luc.edu (E.S.); Elizabeth.Whitham@luhs.org (E.A.W.)

**Keywords:** major depressive disorder, treatment resistance, HPA axis, diabetes, sex hormones, Vitamin D, folic acid, MTHFR, pharmacogenomics

## Abstract

Major Depressive Disorder (MDD) is a highly prevalent psychiatric disorder worldwide. It causes individual suffering, loss of productivity, increased health care costs and high suicide risk. Current pharmacologic interventions fail to produce at least partial response to approximately one third of these patients, and remission is obtained in approximately 30% of patients. This is known as Treatment-Resistant Depression (TRD). The burden of TRD exponentially increases the longer it persists, with a higher risk of impaired functional and social functioning, vast losses in quality of life and significant risk of somatic morbidity and suicidality. Different approaches have been suggested and utilized, but the results have not been encouraging. In this review article, we present new approaches to identify and correct potential causes of TRD, thereby reducing its prevalence and with it the overall burden of this disease entity. We will address potential contributory factors to TRD, most of which can be investigated in many laboratories as routine tests. We discuss endocrinological aberrations, notably, hypothalamic-pituitary-adrenal (HPA) axis dysregulation and thyroid and gonadal dysfunction. We address the role of Vitamin D in contributing to depression. Pharmacogenomic testing is being increasingly used to determine Single Nucleotide Polymorphisms in Cytochrome P450, Serotonin Transporter, COMT, folic acid conversion (MTHFR). As the role of immune system dysregulation is being recognized as potentially a major contributory factor to TRD, the measurement of C-reactive protein (CRP) and select immune biomarkers, where testing is available, can guide combination treatments with anti-inflammatory agents (e.g., selective COX-2 inhibitors) reversing treatment resistance. We focus on established and emerging test procedures, potential biomarkers and non-biologic assessments and interventions to apply personalized medicine to effectively manage treatment resistance in general and TRD specifically.

## 1. Introduction

Major Depressive Disorder (MDD) is a highly prevalent psychiatric disorder worldwide. It causes individual suffering, loss of productivity, increased health care costs and carries high suicide risk. Current pharmacologic interventions fail to produce even partial response in approximately one third of these patients, and remission is obtained in only about one third of them. This is known as Treatment-Resistant Depression (TRD). The Sequenced Treatment Alternatives to Relieve Depression STAR*D trial of 3671 prospectively treated outpatients with non-psychotic depression indicated that remission rates for patients following the first through fourth medication trials were 37%, 31%, 14% and 13%, respectively. Relapse rates increased with each new treatment trial, as did limiting side effects, leading to treatment discontinuation [1]. The burden of TRD exponentially increases the longer it persists, with a higher risk of impaired functional and social functioning, vast losses in quality of life and significant risk of somatic morbidity and suicidality. Patients with residual symptoms of depression at the end of acute treatment are at a higher risk of recurrence, relapse and chronicity. TRD patients are twice as likely to be hospitalized for general medical and comorbid conditions and have increased utilization of health care services and pharmacotherapies, as compared to patients with depression and an acceptable treatment response. Up to 40% of MDD-associated costs in the US are attributable to treatment resistance. The total annual MDD cost burden in the US is estimated to be USD 80 to 130 billion [2]. 

Non-remission and non-response to adequate treatments of depressive illnesses have also been designated as “refractory depression [3,4]. More recently Rush et al. have introduced the designation of “difficult-to-treat depression” (DTD) [5]. Their proposed model stipulates the attainment of sustained remission as the treatment goal. Their model aptly stipulates the assessment of “the many treatable causes of persistent depression” and initiation of corrective action in the hope of reversing the previous negative outcome and attain remission [6]. A more recently published article presents the findings and conclusions of an international group of psychiatrists with expertise in affective disorders from across Europe, US, Canada and Australia who convened to discuss issues relating to DTD [7]. While the group deliberated and presented arguments in favor of one or the other designation and underscored inherent differences in connotation between RTD and DTD, the experts reached consensus that “All depression management should include a thorough initial assessment”. In addition to a regular reassessment, the consensus statement further recommends the employment of a multi-dimensional framework to identify “addressable barriers to successful treatment”. We fully agree with this overall conclusion, and this is the main focus of the present review article. We attempted to present a “map” for psychiatric and non-psychiatric practitioners to follow as a guide not only when managing TRD or DTD but fundamentally as part of an initial evaluation even at the first episode of a depressive disorder and prior to any treatment failures. 

Clinicians and researchers have engaged in intense efforts to unravel the causes of TRD (or DTD) but only with limited success to date. On a positive note, numerous biologic and non-biologic contributory and risk factors have been identified and need to be considered when confronting TRD. Clinical variables that can distinguish patients with TRD from treatment responding MDD patients include psychiatric co-morbidities (e.g., anxiety and panic disorder and social phobia), personality disorder, suicidality, psychosis, substance abuse and dependence, early age at onset, higher numbers of hospitalizations and recurrent episodes. A range of co-morbid medical conditions, notably, endocrinopathies, cardiovascular disease, neurological diseases and vitamin deficiencies, may also contribute to TRD [8]. With respect to the doctor–patient relationship, failure to conduct in-depth assessment of the patient’s depression and the possible developmental and environmental factors that are likely to be contributory, hurried evaluations by the clinician, failure to follow evidence-based guidelines for MDD diagnosis and treatment, use of ineffective doses of antidepressant agents and failure to incorporate psychotherapy can easily lead to labeling the patient’s condition as “pseudoresistance” [9]. The issue of side effects must also be given due consideration, as they can be a significant deterrent to patient adherence to the prescribed treatment. 

Turning to neurobiological risk factors contributing to or being singly responsible for treatment failure or treatment resistance, progress has been made. Such risk factors include polymorphisms in receptor and transporter genes, and in metabolic enzymes responsible for drug transport and metabolic clearance, all of which can impede antidepressant responses. To date, the most promising TRD-associated genes code for the serotonin transporter SLC6A4, presynaptic serotonin autoreceptor 5-HTR1A, catechol-*O*-methyltransferase (COMT), brain-derived neurotrophic factor (BDNF) and the transcription factor CREB1. Neuronal structural alterations and synapse loss, associated with stress and sleep disturbances, alter the density and function of 5-HT receptors and transporters and may cause reduced cognitive abilities and emotional dysfunction [9,10,11]. Last but certainly not least, the role of stress must be seriously considered in managing TRD patients. Excessive and prolonged stress may result in neuronal structural alterations that contribute to the reduced prefrontal cortical and hippocampal volumes seen in patients with depression. Chronic stress causes dendritic atrophy of neurons within the hippocampal formation and medial prefrontal cortex; an enriched environmental exposure during the period of stress can induce prefrontal-hippocampal plasticity [12]. 

In this review article, we focus on parameters and risk factors that have been associated with failure to respond to antidepressant drug therapies, also referred to as treatment resistance. Space limitations preclude an exhaustive review of the individual parameters addressed in this article. Rather, we focus on those tests and interventions that are technically feasible and can be obtained for a large segment of the population in most countries of the world. In a separate section of the article, we also describe additional tests, biomarkers and interventions that are likely to prove widely beneficial once their validity and reliability have been confirmed in future and large-scale studies. Our recommendations are illustrative of the value of practicing personalized medicine rather than engaging in random approaches to manage effectively one of the most serious and prevalent disorders of humanity. 

## 2. Endocrinopathies

### 2.1. HPA Axis

MDD is a multi-system illness, and while many of these interacting systems have not yet been adequately studied, new conceptualizations of the disease have shed light on potential therapy targets. Among these hopeful targets is the HPA axis. The association between stress and MDD has been extensively studied, and we have evidence that stressful life events often precede the onset of a depressive episode. In fact, the majority of individuals have experienced a major life event 3-6 months prior to the onset of their depressive episode [13]. Additionally, persons with MDD exhibit altered psychological and neurological responses to stress and stressful life events [14,15]. The HPA axis has been well documented as both a biomarker and mediator of depression and is actually among the most researched biological systems in relation to MDD [16].

Numerous studies and recent meta-analyses assessing the HPA axis and depression have shown that depression is associated with increased cortisol and that patients with MDD have a diminished cortisol awakening response (CAR) [17,18,19,20]. Chronically elevated cortisol levels are associated with poorer outcomes in the treatment of MDD, leading to the characterization of some patients as “treatment resistant” [21]. Studies suggest that hypercortisolemia is associated with impaired cognitive function and is a risk factor for subsequent MDD in patients that are at risk [22,23,24]. One study investigated the detrimental effects of synthetic glucocorticoid treatment and its association with mental illness, highlighting the importance of acknowledging this crucial pathway when considering the label of “treatment resistant depression”. This study of over 350,000 primary care patients showed that even when the underlying medical illness is controlled for, patients treated with synthetic glucocorticoids are 7 times more likely to attempt suicide, and twice more likely to develop MDD [25]. 

As we recognize and assess the multiple systems at play in the development and recurrence of depression, we should reconsider the term “treatment-resistant”. We know that the HPA axis is implicated in MDD and other stress-related conditions, but there are currently no FDA approved medications for MDD that target the HPA axis. However, diagnostic testing is available to clinicians to assess HPA activity, yet this is not routinely used in the clinical setting to identify patients likely to benefit from such personalized treatment plans [26].

As mentioned above, among the most consistent biological changes in MDD patients is increased plasma cortisol and overall dysregulation of the HPA axis [27,28]. The HPA axis is activated in the presence of a perceived stressor with ensuing release of corticotropic-releasing hormone (CRH) and adrenocorticotropic hormone (ACTH), which stimulates the adrenal gland to release cortisol [29]. Cortisol is essential for increasing our energy level to appropriately deal with stressful situations [20]. Cortisol increases blood sugar by activating gluconeogenesis and glycogenolysis [30]. Among the most important aspects of the HPA axis that is implicated in MDD is the negative feedback loop in which cortisol starts to act as a suppressor of the HPA axis once it is carried back to the hypothalamus and pituitary via the blood stream. As part of this negative feedback loop, cortisol binds to mineralocorticoid and glucocorticoid receptors (MRs & GRs), inhibiting the release of CRH and ACTH. Over time, this also decreases the amount of cortisol released systemically, and the system goes back to homeostasis when the stressor is gone [20].

In other non-psychiatric diseases, such as Cushing’s disease or Addison’s disease, where there is hypercortisolemia and hypocortisolemia, respectively, patients experience severe symptoms, including psychiatric symptoms that require surgical and/or medical treatment [20,31]. We, therefore, recommend that clinicians should routinely assess HPA axis function, especially in MDD patients with MDD and presumptive “treatment resistance”. This is highly important because when other causes of HPA dysfunction can be ruled out, it is critical to identify other causes of chronically elevated or decreased cortisol levels, such as chronic stress.

A normal stress response to a short-term stressor consists of the adequate release of cortisol and ultimately recovery back to homeostatic levels of cortisol once the stressor is terminated. The problem arises with chronic HPA axis activation, which leads to consistently elevated cortisol that fails to return to homeostatic levels. This failure to return to homeostasis leads to chronically elevated cortisol, which ultimately desensitizes glucocorticoid receptors (GRs) [30]. The integrated negative feedback of the HPA axis via GRs at the hypothalamic and pituitary levels is responsible for the downregulation of cortisol secretion. This feedback loop increasingly fails due to a reduction in both the number of expressed GRs and GR desensitization [32]. This ultimately leads to a paradoxical increase in basal cortisol levels, but a decrease in glucocorticoid signaling, which is associated with fatigue, decreased mood and impaired cognitive function [32,33].

If HPA axis dysfunction has consistently been shown to be implicated in MDD, then why is it not part of routine psychiatric evaluation? Multiple methods for measuring HPA function have been utilized; however, results have been mixed. While some studies show that increased cortisol is associated with treatment non-response [19,34], other studies have shown increased cortisol to be associated with improved treatment response [35,36]. These differences need to be rectified, as they could be attributed to various media of cortisol measurement. Cortisol can be measured in plasma, serum and saliva. Another factor that affects cortisol levels is diurnal variation. One last factor to consider with these mixed results in the literature is that cortisol levels change depending on whether they are collected with or without a challenge [28]. 

#### 2.1.1. Measurement

Traditionally the diagnosis of MDD and psychiatric illnesses in general is based mainly on a patient’s subjective symptoms, which are assessed by the clinician in a clinical setting. Unlike many non-psychiatric disorders, the quantification of neuropsychiatric pathology is non-existent. Our goal in the context of personalized medicine is to re-conceptualize the standard of care by addressing the multi-factorial nature of MDD and other psychiatric diseases. Given that the HPA axis can be quantitively assessed, we suggest the routine assessment of patients with respect to HPA dysregulation. 

Salivary cortisol appears to be the most accurate measurement of cortisol because it reflects non-protein-bound cortisol in blood. Compared to urine and plasma cortisol, salivary cortisol is cheaper, less invasive and requires less laboratory resources [20]. One commonality shared by all three collection methods is diurnal variation; therefore, it is essential to remember that one specimen collection is only measuring that moment in time. Cortisol peaks in the early morning and decreases as the day continues [20]. This peak in cortisol is called the cortisol awakening response (CAR), which occurs in all humans and is believed to be used to fuel the human body by transiently activating gluconeogenesis and glycogenolysis [20,37].

As mentioned above, cortisol can be reliably measured, but how do we measure HPA axis function? HPA axis function can be measured using challenge tests. One such challenge test is the dexamethasone-suppression test (DEX), which was among the first tests used to assess stress-related psychiatric disorders [38]. Dexamethasone is a synthetic glucocorticoid that binds to the GR in the CNS, specifically the hypothalamus and the pituitary gland. Normally, administering synthetic glucocorticoids should activate negative feedback and slowly decrease cortisol levels. If there is HPA axis dysfunction, then cortisol levels will fail to decrease and may actually increase. In the case of MDD and potential characterization of TRD, we suggest that this failure to activate the negative feedback is indicative of HPA axis dysfunction, potentially due to receptor insensitivity [20]. Most studies have demonstrated that severely depressed patients often show non-suppression and impaired feedback inhibition by dexamethasone, which is indicative for dysfunction of corticosteroid receptors, especially GR [39,40]. Other challenge tests include the CRH test; the combined DEX-CRH test; and the newest test, the prednisolone suppression test (PST). The PST is the gold standard for assessing HPA axis function [20,41] because it is able to measure both the MR and the GR, unlike the other challenge tests. 

#### 2.1.2. Treatment

The increase in biomarker research and potential treatments hold promise not only in adequately treating MDD but also enhancing treatment responsivity in TRD. Some of these potential treatments are already FDA approved in other fields for other diseases, including anti-inflammatory medications (celecoxib and other cyclooxygenase-2 inhibitors), TNFα antagonists (etanercept and infliximab), minocycline and aspirin [42].

In particular, the altered regulation of ACTH and cortisol secretory activity, along with impaired corticosteroid receptor signaling, have been postulated to underpin depressive psychopathology [20,43,44]. More importantly, in patients where HPA axis abnormalities were not treated, patients were at a much higher risk of relapse or treatment resistance [43], supporting the need to assess the HPA axis in patients that are treatment resistant. There have been interventions using HPA components as targets, including CRH receptor antagonists, GR antagonists and cortisol synthesis inhibitors. In more recent studies, inhibiting the GR chaperone protein FKBP51 with a selective inhibitor has proved effective [43,45].

Other recently studied biomarkers include interleukin 1B (IL-1B), macrophage inhibitory factor (MIF) and oxytocin. IL-1B is implicated in HPA overactivation and, therefore, may also be a potential treatment target for TRD. In addition to the already established literature on cytokine activity in depression, MIF has become a new biomarker and target in MDD [46]. MIF is a hormone with many immunological functions, but it is also a hormone secreted by the anterior pituitary and adrenal gland, after HPA activation. When MIF is released, it acts to oppose the inhibitory actions of glucocorticoids and, therefore, induces steroid resistance. MIF counter-regulates the suppressive effects of glucocorticoids on target immunoinflammatory cells, and through this property, it may be responsible for the induction of steroid resistance [47,48]. Oxytocin is a neuropeptide that suppresses the HPA axis, and recent studies have provided evidence that intranasal oxytocin added to escitalopram may be beneficial in TRD [49].

While medical management holds promise in the realm of MDD, specifically re-conceptualizing TRD, it is crucial to address non-medical aspects of chronic HPA activation. First of all, evidence suggests that HPA axis alterations in depression may be rooted in childhood trauma [50]. A recent meta-analysis showed that patients who experienced childhood trauma, and therefore, potentially chronic activation of the HPA axis, had poorer responses to treatment overall [51]. Therefore, it may be prudent as a clinician to routinely screen for this subgroup of patients with depression and potential TRD.

In patients that have perhaps been screened for childhood trauma/stress and who are exhibiting TRD, we recommend considering a personalized approach both medically and non-medically. This may involve interventions that are focused on trauma. In regard to chronic stress, it may be advisable to direct patients to therapies that focus on interpersonal aspects that are often a source of chronic stress. The large amount of research in this field is extremely hopeful, but it requires clinicians to routinely assess the multi-systemic nature of MDD, to identify potentially treatable causes of treatment resistance. 

### 2.2. Diabetes and Depression

Increasing evidence suggests a bidirectional relationship between depression and insulin resistance, particularly as it relates to Type 2 Diabetes Mellitus (T2DM). Given the growing evidence associating these two common diseases, the comorbidity is continually under-recognized and under-treated. Some estimate that T2DM and depression are twice as likely to occur together [52,53,54]. Concurrent depression and insulin resistance has been shown to increase depressive symptom severity as well as decrease the effectiveness of antidepressant treatment [55,56]. Molecular mediators, such as glutamate and brain-derived neurotrophic factor (BDNF), have been identified as potential mediators of the bidirectional relationship between T2DM and depression [55,57,58]. Considering this essential relationship and the significant disability that T2DM and depression cause independently, let alone concurrently, we urge clinicians to consider the interrelatedness of these two diseases when assessing patients for “treatment resistant depression”. 

Insulin resistance, which may later develop into T2DM, is underrecognized and often undertreated. When combined with depression, these diseases are further under-diagnosed and may cause significantly more impairment, undermining the effectiveness of standard antidepressant treatment. Therefore, we suggest that treatment response to antidepressants may be improved if the underlying insulin resistance is adequately recognized and treated. This suggestion to clinicians is evidence-backed and enables us to reconceptualize the treatment of depression in individuals with concurrent insulin resistance. 

There are multiple ways to approach the treatment of T2DM, including pharmacological and non-pharmacological interventions. Depression and T2DM are hypothesized to share certain pathophysiological characteristics, potentially contributing to their bi-directional relationship. For instance, depression can be conceptualized as a reaction to the complexities of managing a chronic disease, such as T2DM. On the flipside, lifestyle and behavior associated with depression can increase the risk of insulin resistance and potentially T2DM. This may be due to the fact that people with depression may be less inclined to be physically active and may consume diets that are high in refined sugars and saturated fats, which increase the risk of developing T2DM [54,59].

In addition to the abovementioned lifestyle and psychosocial factors, there are also fundamental biological mechanisms shared by T2DM and depression. These mechanisms include the overactivation of the HPA axis and inflammation, each of which is a predisposing factor for the development of diabetes and depression [60,61]. HPA axis dysfunction as mentioned above can ultimately manifest as decreased GR sensitivity, thereby affecting insulin receptor function [61]. Multiple studies have shown that depression is associated with hyperglycemia and, even more importantly, that improved glycemic control helps attenuate depressive symptomology [58,60]. Insulin sensitizers, such as pioglitazone, have been shown to augment antidepressant treatment response, especially in TRD [56,62]. If we can improve insulin sensitivity through lifestyle or pharmacological intervention, we may be able to increase responsiveness to anti-depressants in patients with comorbid depression and T2DM.

### 2.3. Gonadal Hormones

Hyperactivity of the HPA axis inhibits the synthesis and secretion of hormones in the hypothalamus, pituitary gland and gonads; gonadotropin-releasing hormone in the hypothalamus; FSH and LH in the pituitary; and testosterone and estradiol in the gonads [17]. As mentioned in the HPA axis section above, elevated cortisol is among the most robust pathophysiological findings in mood disorders [17]. Stress not only predisposes people to mood disorders but also perpetuates mood disorders, potentially contributing to TRD [63]. Stress, as it relates to the HPA axis and hypercortisolemia, contributes to alterations along the HPG axis, which in turn may facilitate the predisposition, onset and perpetuation of depressive disorders.

After the onset of puberty, the prevalence of anxiety and depression in males and females changes, suggesting an association between mood disorders and sex hormones [64,65]. Many studies suggest that the greater prevalence of depression in women is related to hormonal fluctuations throughout the female life cycle, including menstrual changes, pre and postpartum changes and menopause [64,65,66]. In contrast to females, males have a fairly linear decrease in sex hormones, particularly testosterone, throughout their lifespan. Estradiol and testosterone are gonadal hormones, both centrally and peripherally, that make up the HPG axis and also influence the HPA axis, cognition, metabolism and other critical autonomic functions [17]. Estrogen’s and testosterone’s effects on the HPA axis are mediated via glucocorticoids. Elevated glucocorticoids are associated with mood disorders, heart disease and obesity [64,65]. Given that these hormones are so intricately associated with mood disorders, we propose that physicians objectively assess levels of estrogen, testosterone and other metabolites through simple blood tests. We recommend that before assigning a patient the label “treatment resistant”, the physician should consider adding additional blood tests, such as estrogen, free testosterone, bound testosterone and DHEA, to the routine cell blood count and complete metabolic panel.

Given the increased rates of anxiety and depression in hypogonadal, aging men, it is postulated that androgens and estrogen may be neuroprotective as it pertains to mood disorders. Androgens function via androgen receptors but can also bind to estrogen receptors [67,68,69]. Androgen replacement therapy, specifically testosterone replacement therapy in aging, hypogonadal men not only improves mood but also improves depressive symptoms. As mentioned previously, the literature suggests that the HPA axis and HPG axis are closely intertwined, and in fact, it has been shown that testosterone and cortisol inhibit the release of each other [67,68,69]. If the HPG axis negatively regulates the HPA axis, then the HPG axis may play an essential role in decreasing TRD, since chronic high levels of glucocorticoids are a key contributor to the development of these disorders [67,70].

DHEA is a precursor to progesterone, which is a precursor to the gonadal hormones, testosterone and estradiol. Additionally, DHEA also acts independently on the HPA axis, strengthening the association between the HPA and HPG axis [71]. Low DHEA levels have consistently been associated with increased depressive symptoms [71]. In fact, treatment with DHEA as an antidepressant therapy has shown some success [72]. Progesterone, a downstream metabolite of DHEA, and its own metabolites act as neurosteroids and are tightly associated with mood disorders. Progesterone and its metabolites are allosteric modulators of the GABA A receptor. This means that progesterone and its metabolites can inhibit excitatory signals that would otherwise result in feelings of anxiety and fear. Another mechanism proposed in the literature to explain the neuroprotective properties of progesterone and its metabolites relates to the glutaminergic system since these neurosteroids act as NMDA and AMPA receptors [73].

Allopregnanolone is a downstream metabolite of progesterone, which is itself derived from pregnenolone in the cholesterol synthesis pathway. Pregnenolone’s alternative metabolic pathway includes DHEA, a precursor to testosterone and estradiol. In males, testosterone is recommended as replacement therapy for individuals with primary hypogonadism as well as hypogonadotropic hypogonadism. Studies have suggested that treatment with certain antidepressants (SSRIs) increases the levels of progesterone and allopregnanolone, which may decrease depressive symptomatology [72,74,75]. 

#### Possible Treatments

A recent review of the literature regarding testosterone use for depression revealed that testosterone augmentation has the largest effect in middle-aged (<60 years old) hypogonadal males with MDD. Testosterone monotherapy was found to be the most useful in patients with dysthymia or minor depression. This same study did not find any significant differences between oral testosterone, testosterone gel, oral dehydroepiandrosterone or intramuscular testosterone use. The same study showed that in these men, depression was associated with a decrease in total or plasma concentrations of testosterone [76]. In another study, hypogonadal men with MDD who were unresponsive to SSRIs showed improved response after testosterone replacement therapy (TRT) [77,78].

Transdermal estrogen treatment has been shown to improve ratings on depression rating scales. In other studies, premenopausal females who were taking oral contraceptives (OC) were found to have decreased rates of mood disorders in the transition to menopause [70]. In another study, longer use of oral contraceptives from menarche to menopause showed a reduced risk of postmenopausal depression [79]. The results of the above studies suggest that ovarian hormones are protective against mood disorders. Estrogen and progestin hormone replacement therapy and allopregnanolone (a progesterone metabolite) are the best studied ovarian hormonal interventions for depression in women [70,71]. In fact, studies have found moderate evidence of efficacy of estrogen replacement therapy or hormone replacement therapy for perimenopausal or early postmenopausal women with major depression and physical symptoms of menopause. More recently, a formulation of the hormone allopregnanolone was approved for the use of postpartum depression. Intravenously administered Brexanolone is among the first FDA approved non-steroidal hormones for use in psychiatric illness [71,72].

Because most antidepressants have adverse side effects, these side effects are the major reason for medication discontinuation. An important side effect of most antidepressants is sexual dysfunction. SSRIs are first line treatment for depression; however, they can cause sexual dysfunction in approximately 1/3 of patients. Testosterone’s positive effect on both mood and sexual function makes TRT a great treatment strategy, either as augmentation and/or monotherapy for males with hypogonadism. Surprisingly, even after TRT is discontinued, the beneficial effects of TRT in hypogonadal men have been shown to continue. This is hopeful for hypogonadal, depressed men considering they may experience long-term benefits from just a short trial of TRT [74].

Recommendation: perform relevant hormonal blood tests and take corrective action.

## 3. Vitamins

Vitamin deficiencies have long been known to cause and/or contribute to medical and psychiatric illnesses. More recently, the role of Vitamin D has been more extensively studied in psychiatric disorders, notably, depressive illness. Vitamin D has also been shown to afford an anti-inflammatory action, which, as we will discuss later in this article, also relates to the pathophysiology of depression.

### Vitamin D

Vitamin D comprises a group of secosteroids and is centrally involved in the regulation of calcium and phosphorus metabolism and immunomodulation. Vitamin D deficiency is a common problem globally in children and adults, estimated to impact 1 billion people worldwide [80]. A growing body of evidence supports Vitamin D’s role in modulating illness, including psychiatric, autoimmune, infectious and cardiovascular diseases. Of particular interest is the role Vitamin D plays in the pathophysiology of depression, supported by research findings of the correlation between low levels of Vitamin D and depression, as well as with a higher burden of depressive symptoms among those with concurrent deficiency [81,82,83,84,85,86]. This has been noted in elderly populations, who have reduced skin synthesis of Vitamin D3 due to a reduction in 7-dehydrocholesterol [80]. Vitamin D has been implicated across different kinds of depression, including with perinatal onset, seasonal pattern or bipolar depression [87,88,89].

Vitamin D has been posited to have many functions beyond controlling blood levels of calcium through the regulation of gene expression, including the regulation of proliferative and apoptotic activity, immunomodulatory activity, insulin secretion, interaction with the renin-angiotensin system and neuroprotective functions. In particular, the evidence linking Vitamin D and the proinflammatory cytokine theory of depression is compelling, with an association between the severity of depression and increased concentrations of inflammatory markers. This is underscored by direct evidence of calcitriol modulation of proinflammatory cytokines both in vitro and in vivo. Relevant also to a potential causative role of Vitamin D and mood disturbance is the presence of Vitamin D receptors in the cortex, limbic system and cerebellum, with ample evidence linking Vitamin D and brain function [89,90].

The causal role of Vitamin D deficiency in depression, and the efficacy of supplementation to correct this deficiency and thus ameliorate depression, remains somewhat controversial, with studies showing mixed results [91,92,93,94,95]. This is in part due to limitations in study design in assessing for the presence of baseline Vitamin D deficiency, rather than enrolling and offering supplementation to participants indiscriminately. A second concern is validating the presence of an index depressive episode prior to treatment, as in several studies, patients with low depressive scores have been included without a clinical diagnosis of major depression. Some studies have offered supplementation to assess prevention, such as a recent randomized controlled clinical trial of Vitamin D supplementation in over 18,000 older adults without index depressive episodes, which was not found to decrease the incidence of depressive episodes [96]. A third study design concern pertains to treatment, wherein effective treatment dosing is offered over an appropriate interval of time. A recent meta-analysis of four randomized controlled trials (RCTs), of which two enrolled participants with lab proven Vitamin D deficiency showed low levels of heterogeneity and moderate effect size, suggested a clinical benefit of Vitamin D supplementation [95] among depressed patients. Some studies have suggested that the effect is comparable to that of typical antidepressant medication treatment [94]. There is a need for additional RCTs among those who are depressed and Vitamin D deficient to further substantiate these findings [91]. Given the support for the hypothesis that correcting Vitamin D deficiency decreases concomitant depressive symptoms, patients with TRD are a population of particular interest for the investigation of potential deficiency and treatment.

The assessment of Vitamin D deficiency and subsequent treatment is both safe and economical. Studies of Vitamin D supplementation show no adverse effects even at high doses of up to 10,000 IU daily, and doses of 800 IU are generally sufficient to reach a 25(OH)D level of at least 50 nmol/L (or 20 ng/mL). Additionally, treatment does not require the monitoring of serum or urinary calcium or renal function [97]. We strongly encourage the measurement of Vitamin D blood levels and supplementation commensurate with the magnitude of the abnormality among those individuals with TRD. 

Recommendation: measurement of blood levels of specific vitamins, notably, B9, B12 and D, and supplement as appropriate.

## 4. Folic acid

### Folic Acid, L-methylfolate, MTHFR

**Figure 1:**
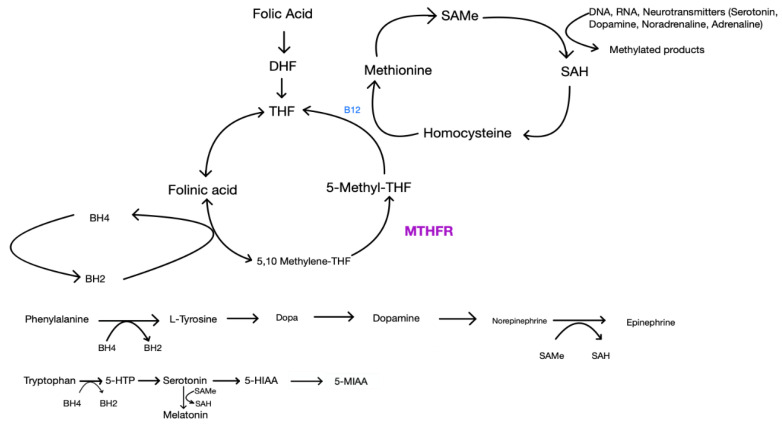
Folic acid pathway and other critical pathways involved in neurotransmitter synthesis.

A growing body of literature suggests that one-carbon metabolism may moderate antidepressant treatment response [98]. The one-carbon cycle includes agents such as folate, L-methylfolate, S-adenosylmethionine (SAMe), vitamin B6, vitamin B12 and homocysteine. While the literature suggests that deficiencies in each of these components can be independently associated with depression, folate levels in particular are the most common [99]. Folate, otherwise known as vitamin B9, comes in 2 forms: as dihydrofolate (in foods) or in a synthetic form (supplements). The first step in this one-carbon metabolism is the conversion of synthetic or dietary folate to 5-methyltetrahydrofolate (L-methylfolate) via methylenetetrahydrofolate reductase (MTHFR) [100,101,102,103,104,105,106]. L-methylfolate (also known as levomefolic acid or 5-methyltetrahydrofolate) is the active form of folate or folic acid and plays a critical role in the synthesis of neurotransmitters. Methionine synthetase then uses the active form, L-methylfolate, as a methyl donor to convert homocysteine into L-methionine. Vitamin B12 helps carry out this reaction. Methionine then reacts with ATP to form SAMe. SAMe acts as a methyl donor, similarly to L-methylfolate, and plays a critical role in the methylation of monoamines and, therefore, the synthesis of neurotransmitters via the tetrahydrobiopterin (BH4) pathway and the hydroxylation of phenylalanine and tryptophan [100,101,102,103,104,105,106,107]. The folic acid pathway is illustrated in Figure 1 below.

**Figure 2:**
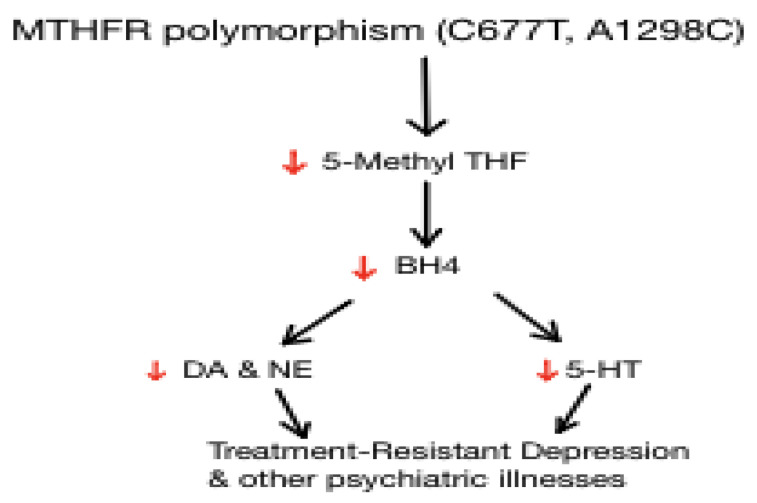
Downstream effects of MTHFR polymorphism and implications on neurotransmitter synthesis. MTHFR polymorphisms lead to decreased synthesis of L-methylfolate, leading to decreased levels of dopamine, norepinephrine and serotonin.

This is the normal synthesis of several neurotransmitters. MTHFR is the enzyme that converts 5,10 Methylene-THF to the active form, 5-Methyl-THF. The effects of MTHFR polymorphisms are shown in Figure 2.

Numerous studies have demonstrated the association between depression and folate deficiency [98,99,108,109,110,111]. L-methylfolate, the active form of vitamin B9, is the only form that can cross the blood–brain barrier [112]. Low levels of L-methylfolate are associated with multiple neuropsychiatric diseases, including MDD, schizophrenia and Alzheimer’s. Studies also show that individuals with a deficiency may exhibit inadequate response to antidepressants, perhaps leading to the label of “treatment-resistant” [108].

Studies have shown the effectiveness of both folic acid and L-methylfolate as both a monotherapy and as an adjunctive therapy, suggesting the importance of assessing folate status in patients that appear to be “treatment resistant” [99,108,110,111]. In fact, L-methylfolate is among the only medical foods licensed by the FDA for the treatment of depression [113,114]. In a review article, Papakostas et al. [21] examined 4 trials that used L-methylfolate as monotherapy; two were open trials and two were double-blind trials that investigated L-methylfolate versus antidepressants [109].

In regard to investigating the use of L-methylfolate as an adjunctive therapy, the first trial performed was a double-blind placebo-controlled trial in which 24 patients with MDD and RBC folate deficiency were given 15 mg/day of L-methylfolate as an adjunct to their antidepressant treatment. At 3- and 6-months postintervention, patients that had received L-methylfolate had a greater improvement compared to placebo [115,116]. In a more recent meta-analysis, the authors concluded that the inactive folic acid cannot be firmly recommended, but that the active form, L-methylfolate and SAMe, can be recommended as an adjunctive treatment for MDD. Randomized, controlled trials investigating the adjunctive use of L-methylfolate in adults with TRD have shown partial response to antidepressants. More specifically, those with a polymorphism in the MTHFR gene, leading to decreased conversion to the active form, may benefit from supplemental L-methylfolate therapy [117]. This recommendation is not only supported in multiple meta-analyses but also through the literature; agents of the one-carbon cycle, such as folic acid, L-methylfolate, Vitamin B6, Vitamin B12 and SAMe, are necessary for the methylation of monoamines and, therefore, the synthesis of neurotransmitters that mediate MDD and treatment response [116,118].

While investigating the role of folate deficiency in depression is important, it is also essential to consider the MTHFR gene; individuals may have genetic polymorphisms that affect the conversion of synthetic or dietary folate to L-methylfolate, the biochemically active form. If someone is homozygous for the T variant (TT), studies suggest that they have about 30% of the enzyme activity of people with the wild-type (CC) variant [100,101]. Heterozygous (CT) individuals have about 65% of the enzyme activity of CC individuals. In Caucasian North Americans, 8–20% of the population has the TT genotype. One meta-analysis of 10 studies found a small but significant increase in depression in people with the TT genotype [100,101]. Because MTHFR converts folate into L-methylfolate, the active form, if an individual has a genetic polymorphism that decreases or inhibits this conversion, it is reasonable to assume that this population may be more at risk for MDD and perhaps treatment-resistant MDD. Furthermore, this finding highlights the need for the administration of L-methylfolate, rather than folic acid, so that the defective enzyme can be bypassed.

Another aspect that cannot be overlooked is the integral function of the B vitamins. The B vitamins play critical roles as cofactors in cellular cycles, such as the methionine and folate cycles [119]. B vitamins are also important for DNA methylation and the regeneration of methionine for the folate cycle via clearance of homocysteine [119]. The interrelatedness of these pathways helps demonstrate why deficiencies in one or many of the substrates can limit the pathways. Another critical factor in the folate and methionine cycles is homocysteine [120]. When one nutrient or reagent is missing, this can result in a defective cycle, leading to hyperhomocysteinemia. Hyperhomocysteinemia has been shown to increase risk for poor mood; one study showed that up to 30% of depressed patients have increased homocysteine levels [101,121]. Because of the interrelatedness between these pathways and given that B vitamins are necessary cofactors in the synthesis and regulation of neurotransmitters, such as dopamine and serotonin, we can, therefore, conclude that B vitamins are also implicated in mood regulation, more specifically MDD. Studies have addressed the mood effects of B vitamins in patients with MDD that are also medicated and have shown improvement. While there is no conclusive evidence, this point helps us establish proper strategies in assessing patients that are labeled as “treatment-resistant” [101,107,122,123,124,125]. 

As mentioned above, the interrelatedness of these cycles requires investigating multiple substrates of these cycles. One substrate that has also been studied is SAMe, which is also implicated in homocysteine metabolism. It was originally discovered in 1952 and functions in the synthesis of hormones and neurotransmitters (serotonin, dopamine and norepinephrine) through the homocysteine cycle. [126,127,128,129,130,131]. L-methylfolate is similar to SAMe, in that it is also used as an enzyme substrate in the biopterin cycle to produce monoamines and subsequently neurotransmitters. SAMe concentrations are deficient in a wide variety of neurological and psychiatric disease states, such as in Alzheimer’s dementia, Parkinson’s disease and MDD [131] Studies suggest that oral and parenteral administration of SAMe crosses the blood–brain barrier, which is similar to the case of L-methylfolate. Over 50 clinical trials of varying types have evaluated the use of SAMe in the treatment of depression. A meta-analysis in 2002, which included 28 studies looking at the use of SAMe in the treatment of MDD, concluded that compared with placebo, patients treated with SAMe showed an improvement of 6 points on the Hamilton Rating Scale of Depression compared to placebo. Amongst systematic studies investigating complementary therapies for depression, one randomized control trial showed a significant effect of SAMe as an adjunct to standard antidepressants [132].

Recommendation: perform measurement of blood level folic acid and genomic test for MTHFR, preferably as part of a more comprehensive pharmacogenomic profile as available on the market in your country. 

## 5. Pharmacogenomics

One tool that psychiatrists have utilized over the last decade to help personalize treatment decisions is pharmacogenomic (PGx) testing [133]. PGx testing utilizes genomic sequencing to determine the pharmacokinetic, immune-related and pharmacodynamic effects of variants in specific genes that can affect an individual’s response to medications. Evidence in support of PGx testing in psychiatry has been obtained in individuals with depression who had failed at least one medication. Two meta-analyses showed that patients whose treatment was guided by PGx testing resulted in improved remission of symptoms compared to treatment as usual [134,135].

Therefore, PGx testing may be a valuable tool for clinicians in guiding and personalizing medication decisions [136]. Earlier studies had shown promising results [137,138,139,140,141,142]. More recent and controlled studies have added clinical validity to the severity of gene-drug interactions (GDI) in guiding medication decisions and improving outcomes [143,144,145]. A meta-analysis for one individual commercial test also showed significantly greater outcomes in individuals with depression compared to treatment as usual [146].

In spite of encouraging and promising studies reported to date, pharmacogenomic testing has not yet been routinely adopted by clinicians as an approach for improving outcomes in patients with MDD and TRD. Many remain skeptical or puzzled about such tests. One primary reason for this is that previously available tests have not yet been able to convey which medications are statistically shown to be most effective. Underlying barriers have included small numbers of genes and variants, small sample sizes and short durations for follow-up. While much work remains to be conducted, test shortcomings are being addressed. Combinatorial pharmacogenomic (PGx) algorithm advances are progressively integrating and evaluating multiple pharmacokinetic (PK) and pharmacodynamic (PD) genes. This approach is based on the fact that most medications are not cleared by a single detoxification pathway, different pathways are more important than others and mutations even in minor pathways can exert negative effects. A combinatorial pharmacogenomics test was deployed in the evaluation of 1167 patients with MDD and prior inadequate response to antidepressants, in a blinded, 24-week trial. In this previously treatment-resistant population, guided pharmacogenomic testing significantly improved treatment response (26% vs. 20%; *p* = 0.01) and remission (15% vs. 10%; *p* < 0.01) when compared with unguided treatment as usual [144]. 

As clinicians pursue precision health biomarkers, among the conceptual barriers that will need to be understood are that simple cheek swab results do not compellingly determine which antidepressant will be “congruent” or most effective for a patient. Emerging data now show, however, that such tests aid in conveying which medications may be incongruent choices and incorrect for a given patient, and thereby secure the attainment and perpetuity of response and remission. The use of knowledge of a patient’s genetic makeup (either large discreet genes or SNPs) definitely assists the clinician in making more informed choices in medication selection and thereby personalizing treatment. Such choices must always be made in the context of clinical judgment and relevant patient factors. Ultimately, it is the clinician who knows the patient better than any set of lab results.

Recommendation: Pharmacogenomics testing is strongly recommended. Continued use of substances known to interact with pharmacogenomic and pharmacokinetic parameters must be strongly discouraged, and where indicated, referral of the patient to rehabilitation services to achieve permanent detoxification and abstinence should be considered. 

## 6. The Possible Role of *5-HTTLPR* in Neuroprogression in Depression

### Serotonin-Transporter-Linked Polymorphic Region (5-HTTLPR)

**Figure 3:**
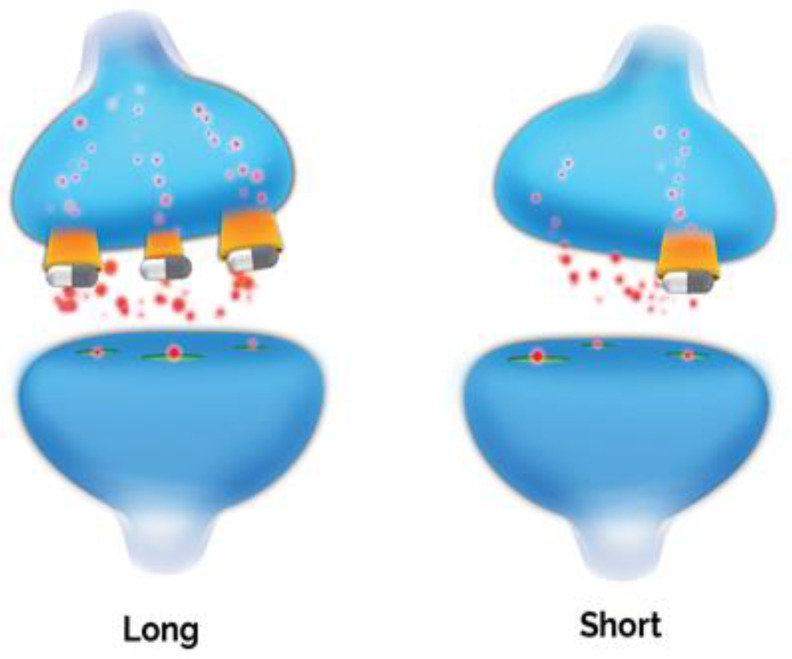
SLA6A4 Alleles. The SLC6A4 promoter variant is among the best studied polymorphisms in psychiatry. Multiple meta-analyses confirm significantly lower rates of response and remission in S/S and L/S individuals.

The serotonin transporter (5HTT), which recycles released serotonin from the synaptic cleft regulating its synaptic levels, is a central player in serotonin homeostasis; it regulates the magnitude, duration and spatial distribution of serotonergic signals, thereby fine-tuning serotonergic transmission [147,148]. The ~31 kbp serotonin transporter gene (*SLC6A4*) is located on chromosome 17 (17q11.1-17q12) consisting of 14 exons. Several variations of the serotonin transporter gene have been studied, with *5-HTTLPR* (serotonin transporter linked polymorphic region) being the most widely investigated candidate polymorphism in psychiatric genetics. *5-HTTLPR* is a 44-base pair insertion deletion polymorphism located in the promoter region of *SLC6A4* (Figure 3). It has two common variants with functional impact; the long (L) allele is associated with more efficient transcription and a higher number of serotonin transporters expressed on neurons, while the short (S) allele is associated with reduced synaptic serotonin reuptake [147,149]. The distribution of the two most common variants shows significant ethnic and geographical differences, with the frequency of the S allele being approximately 42% in Caucasians but up to 78% in Asians, and SS genotype frequency is 21.6–28.3% in Caucasians and 55.6–60.0% in Asians [150,151]. 

*5-HTTLPR* was initially associated with several psychiatric disorders, including those along the affective spectrum, anxiety disorders, substance abuse and personality traits involved in impulsivity, anxiety and stress [152]. Following initial reports of an association between its *5-HTTLPR* variation and anxiety-related traits in the last decade of the last century [149], and results from a longitudinal study suggesting that those who carry the S allele are at an increased risk for depression when exposed to more severe stress [153], there have been many successful and failed replication attempts. The latest and largest meta-analysis concluded that in general there is no robust association between *5-HTTLPR* and depression, either as a main effect or in interaction with environmental events, and if any interaction exists, it must be modest in size [154]. Furthermore, GWAS studies [155,156] also indicate that previously considered candidate genes do not play a role in the genetics of major depression. Several individual studies, however, still suggest a role for 5-HTTLPR in depression, especially in interaction with specific types of stressors; it is hypothesized that there may be a significant association, but it is masked by the lack of differentiation between distinct types of stressors, which may act via divergent genetic pathways [157,158,159]. 

By contrast, unlike in the case of depression, studies of *5-HTTLPR* variation in association with antidepressant responses have been more consistently positive, and *5-HTTLPR* appears to be associated with antidepressant efficacy in Caucasian patients as evidenced in large meta-analyses [160,161,162]. A recently published meta-analysis of 49 studies provided further support that amongst Caucasian patients, those carrying SL and LL genotypes were more likely to respond to or achieve remission with SSRI therapy, while there was no significant effect of these genotypic variants with other antidepressant groups or antidepressants with mixed modes of action. Additionally, no significant association effect emerged in Asian populations [151]. An effect of gender was also found in Caucasian patients with higher remission rates in female L allele carriers [162], although previous studies reported opposite sex associations [163,164]. Age; age at first onset; subtype of depression; course, including number of previous episodes; or seasonality may also have an effect on antidepressant response as a function of *5-HTTLPR* genotype. Anxiety-related personality traits, including neuroticism or harm avoidance, should also be considered [152,160,161,165]. The *5-HTTLPR* genotype was associated with adverse effects of antidepressants. A recent systematic review indicated that reports of increased risk of adverse reactions in Caucasian samples are controversial [166]. However, the S allele was found to significantly increase the risk of antidepressant-induced mania and be positively associated with gastrointestinal side effects [166]. In general, the S allele appears to be associated with increased burden of SSRI-induced adverse reactions [166,167] in Caucasian populations, with weaker evidence in Asian samples.

In spite of the positive association with antidepressant response and remission in Caucasian populations in large meta-analyses, *5-HTTLPR* explains only 3.2% of the variance of antidepressant efficacy [161]. This effect is also influenced by other genes, gene x gene and gene x environment interactions [168], more specifically in conjunction with *5-HTTLPR* and SSRIs [169,170], further obscuring the potential predictive role of *5-HTTLPR* genotyping in predicting SSRI response. Therefore, prior testing of *5-HTTLPR* genotype before initiating SSRI or other antidepressant treatment may not be clinically useful at this point, although *5-HTTLPR* may still be one candidate for a multiallelic test useful to guide personalised antidepressant choice in precision medicine not only for predicting the likelihood of response and adverse effects but also possibly influencing illness course and lowering the risk of depression becoming treatment-resistant or chronic.

*5-HTTLPR* has also been associated with factors directly or indirectly involved in neuroprogression, including epigenetics and gene methylation. Recent studies reported epigenetic mechanisms modifying genes by means of environment interactions and therby influencing the role of the *SLC6A4* gene in psychiatric disorders [148]. The *5-HTTLPR* genotype appears to influence *SLC6A4* methylation with increased methylation in those carrying the S allele [171,172,173,174,175,176]. An association between *SLC6A4* methylation, *5-HTTLPR* genotype and stress sensitivity has also been reported [177,178]. 

Thus, while there is no evidence on the direct involvement of *5-HTTLPR* in neuroprogression to date, *5-HTTLPR* may be involved in stress reactivity leading to depression, as well as in influencing the course of depressive illness, and also in response and remission with serotonergic antidepressants. In summary, by influencing the risk of poor response or treatment resistance, the *5-HTTLPR* genotype in combination with other genetic markers may provide a tool for slowing down or arresting the neuroprogressive course of depression. 

Recommendation: establish if the non-responding patient is homozygotic for the S allele and avoid the use of a SSRI, if other options are available and appropriate.

## 7. Immune System

### 7.1. Inflammation and Depression

Psychoneuroimmunology is the science of immunology, as it relates to psychiatric and neuropsychiatric disorders. It is a relatively young field, having come of age only recently, but the progress that has been made just in the past three decades has exceeded all expectations. The field has opened up new horizons in our understanding of the complex interrelationships between the immune and nervous systems or, as it is otherwise referred to, the brain–immune interaction. Hitherto unknown biochemical pathways have been identified, and their complex interactions with neurotransmitters and immune mediators present opportunities for innovative research and the identification of new targets for drug development. Biomarkers are being established that hold great promise for more precise diagnostic classification of psychiatric disorders, but also for an understanding of the high co-morbidity between specific psychiatric disease entities and a host of medical and neurological diseases. The complex co-morbidity between psychiatric disorders, notably, depressive and anxiety disorders, and cardiovascular, cerebrovascular and neurological disorders can now be better understood thanks to breakthroughs in psychoneuroimmunology. Immune biomarkers, neurotrophins and antibodies enable the prediction of response and understanding of treatment resistance. Imaging techniques of increasing sophistication hold great promise for the visualization of aberrant connectivity and dysfunctional brain circuitry. The goal of practicing personalized psychiatry is now closer to becoming reality than ever before in the history of our specialty. 

The monoamine theory of depression ushered in a new era in psychiatry and has dominated the field for decades. It remains instrumental in the development of innovative therapeutic agents that have been effective, in spite of serious drawbacks, low rates of response and remission and numerous side-effects. Multiple hypotheses have been formulated in an effort to unravel the elusive pathophysiology of depressive illness. In this section, we focus on the cytokine (or macrophage) hypothesis. The cytokine theory of depression was originally formulated by Smith as ”The Macrophage Theory of Depression” and later expanded by Ur et al. [179,180]. The theory postulated that psychological stress, in conjunction with genetic factors, increases cytokine production and leads to depressive symptoms when specific neurobiological systems are affected, such as the HPA axis and serotonergic transmission. A chronic proinflammatory status reflected in elevated circulating levels of proinflammatory cytokines may thus be central to the pathophysiology. The proinflammatory status has been associated not only with MDD and bipolar disorder (BD) but also with atypical depression and posttraumatic stress disorder, and the reader is referred to excellent reviews on the subject [181,182,183,184,185,186,187,188]. Peripheral measures of pro and anti-inflammatory cytokines, commonly referred to as “inflammation biomarkers”, demonstrate variable increases in proinflammatory cytokines and C-reactive protein (CRP). Patients with a depressive disorder have been shown to have increased peripheral blood levels of CRP [189]. It is possible that a chronically elevated level of CRP is a key contributor to TRD. 

### 7.2. C-Reactive Protein (CRP)

CRP is an acute-phase reactant and an early indicator of infectious or inflammatory conditions. CRP modulates the host’s immune response against pathogens and sterile inflammation related to surgery, trauma, psychic stress, myocardial infarction and neoplastic diseases [190]. Traditionally, CRP and high sensitivity CRP (*hs*CRP) have been used as a measure of non-psychiatric conditions. CRP might exert both anti-inflammatory and pro-inflammatory actions. The pathogenesis of psychiatric disorders is not fully understood, but studies suggest that low-grade systemic inflammation contributes to the development and progression of psychiatric disorders. CRP is among the most extensively studied biomarkers in psychiatric patients. Although accumulating evidence suggests that elevated inflammatory markers are associated with psychiatric disorders, there is no identifiable disease-specific or episode-specific biomarker to date. However, CRP might be considered a “psychiatric biomarker”, which may alert clinicians about the adverse effects of medications, a patient’s cardiometabolic status and co-morbidities, and may predict clinical outcomes and optimal treatment selection.

Both inflammatory and anti-inflammatory effects of CRP have been documented previously [191]. The inflammation hypothesis is considered as one of the key mechanisms in the pathophysiology of mood and anxiety disorders [192]. Clinical studies have reported fairly consistently that circulating inflammatory markers, such as CRP and several cytokines, are related to increased risk for depression [193,194]. The prevalence of low-grade inflammation (CRP > 3 mg/L) in depression is 27%. The prevalence of elevated CRP (>1 mg/L) in depression is 58%. Higher levels of CRP at baseline are associated with an increased risk of depression in subsequent follow-ups and systematic review and meta-analysis [195]. The concentrations of circulating CRP are higher in patients with acute depression compared with the control group [196]. A significant association was noted between depression and CRP [197]. Overall CRP levels are significantly elevated in patients with bipolar disorder compared with controls. The increase was higher in manic and euthymic patients. CRP levels were not related to lithium or antipsychotic medication. Meta-analysis [198] showed that CRP levels were increased in bipolar disorder, regardless of mood state. The CRP levels were not related to symptom severity during depression and mania. After treatment for mania, CRP levels moderately decreased, but this decrease was slight after treatment for depression [199]. A recently published article by Moriarity et al. underscores that CRP is uniquely related to fatigue and changes in appetite, suggesting that these phenotypic manifestations of depression should be assessed, as they may be responsive to anti-inflammatory treatments [200]. Similarly, Turkheimer et al. demonstrate a strong association between increased peripheral inflammation as indicated by elevated levels of CRP and reduced blood-to-brain and blood-to-CSF transfer of TSPO-PET radiotracers. The potential consequences of this finding are discussed in their article [201]. 

### 7.3. Cytokines

The role immunological and inflammatory factors may play in psychiatric disorders was hypothesized almost a century ago. It has been supported and confirmed by accumulating evidence linking infections, autoimmune disorders and elevated inflammation biomarkers to the pathophysiology of schizophrenic, affective and stress-related disorders. Recent studies have identified alterations of blood cytokine networks and increased microglial activity in many such patients. Cytokines are small proteins released by cells that act via receptors to regulate the growth, maturation and responsiveness of particular cell populations. Cytokines are peptides, proteins, or glycoproteins. They are produced by a wide variety of immune and non-immune cells and subsume interleukins (ILs), chemokines, interferons (IFNs), lymphokines, monokines and growth factors.

Cytokines play a key role in regulating the immune system, and cytokine pathways have been used as suitable targets for therapeutic interventions, notably, tumor necrosis factor (TNF), IL-1, interleukin 2 receptor (IL-2R), IL-6R, IL-12, IL-23 and receptor activator of nuclear factor-kB ligand [202]. 

### 7.4. Anti-Inflammatory Treatments in Affective Disorders

The enzyme cyclooxygenase-2 (COX-2) converts arachidonic acid to prostaglandins, which are precursors for the synthesis of pro-inflammatory cytokines. COX-2 inhibitors have been increasingly utilized as augmentation strategies to reduce inflammatory burden. Celecoxib (CBX) belongs to the group of non-steroidal, anti-inflammatory drugs (NSAIDs), a large family of drugs that inhibit one or both isoforms of COX (COX-1 and COX-2) on the arachidonic acid cascade, thereby reducing downstream prostaglandin synthesis. In preclinical studies, CBX enhanced 5-HT output and potentiated the effects of reboxetine and fluoxetine on norepinephrine and 5-HT output [203,204,205]. The inhibition of COX-2 enhanced the accumulation of arachidonic acid, which is associated with preferential shunting towards neuroprotective eicosanoids [206]. 

The new generation of NSAIDs selectively inhibit COX-2, which is induced by inflammatory cytokines acting on neurons, particularly in the hippocampus and cortex [207]. CBX has emerged as the prime candidate from amongst marketed NSAIDSs to test the hypothesis that the inhibition of COX-2 should result in a reduction in PGE2 synthesis, particularly in the brain. Based on experimental evidence that CBX reduces the stress response and cognitive deficits in rats [208] and also the behavioral and immune changes in the olfactory bulbectomized rat model of depression, a series of studies were undertaken that we refer to here [188]. 

A number of open, and randomized, double-blind trials of COX inhibitors have been reported to date. An earlier open study showed the superiority of adjunctive aspirin to fluoxetine [209]. The adjunctive use of CBX has been positive in that it enhanced the response of antidepressants with overall good tolerability and safety. Agents used concurrently have included fluoxetine, reboxetine, sertraline and mood stabilizers [210,211,212,213]. The adjunctive treatment of CBX with an antidepressant has also been shown to be beneficial in BD patients, as demonstrated by the study of Nery et al. and our own study with treatment-resistant bipolar depressed patients given escitalopram and CBX [214,215]. 

Recommendations: Investigate the presence of an inflammatory process anywhere in body and take corrective action to reduce or eliminate this source of inflammation. Measurement of *hs*CRP may be a useful marker of an inflammatory process in the body. Use of an anti-inflammatory agent, preferably a COX-2 inhibitor, may assist in converting TRD to treatment response. 

## 8. Ancillary Treatments

### Non-Biologic Factors and Combined Treatment 

Psychological evaluations of patients with MDD without personality disorders have found a preponderance of harm avoidance (anticipatory worry, fear of uncertainty, shyness and fatigability) in patients with TRD compared to those with disease remission. In contrast, those with TRD had lower scores in reward dependence (sentimentality, attachment and dependence), self-directedness (responsibility, purposefulness, resourcefulness, self-acceptance and congruent second nature) and cooperativeness (social acceptance, empathy, helpfulness, compassion and pure-heartedness) compared to remitted study subjects when assessed utilizing a dimensional psychological model, the Temperament and Character Inventory (TCI) [216]. Additionally, patients with TRD tend to score higher in neuroticism and lower in extraversion, openness and conscientiousness [217]. These temperamental and characterological features may be risk factors for TRD, relating either primarily to TRD or representing a secondary effect of depression itself as a consequence of the long illness duration. 

Comorbid personality disorders do not improve outcomes in MDD [218], and have been shown to predict poor response [219,220,221,222]. Estimates of prevalence vary widely, from 14% to 85%, with depressive symptoms potentially clouding personality assessment [223]. Personality may represent a vulnerability with variability in its trajectory over time, influenced by life events [224]. Patterns of personality have historically been described as underlying mood disorders. Emil Kraepelin identified four affective temperaments: depressive, manic, irritable and cyclothymic [224]. The association between personality and MDD is complex, with evidence from twin studies suggesting that though they share genetic and environmental risk factors, they are distinct entities, as MDD risk is influenced by unique genetic contributions [225]. Personality and temperament should be assessed in all patients with MDD, and particularly TRD, and addressed in treatment. 

Psychotherapy as an adjunctive therapy, or combined treatment, should be considered in TRD. A large randomized controlled trial of cognitive behavioral therapy (CBT) as an adjunctive treatment to usual care in patients without response to initial pharmacotherapy showed three-fold increased odds of response at 6 months compared to usual care alone [226]. These findings were similar to a study of cognitive behavioral-analysis system of psychotherapy (CBASP) in combination with nefazodone for chronic depression [227], which showed a substantial benefit in response (85% by 12 weeks) compared with the nefazodone group (55%), and the psychotherapy only group (52%). In this study of CBASP, the advantage of combined treatment was seen following the initial four weeks of treatment, suggesting additive independent effects. In contrast, the REVAMP trial examined medication treatment alone versus combination treatment with adjunctive psychotherapy, either CBASP or brief supportive psychotherapy (BSP) in chronic depression, and no difference in outcome was found between groups [228], though the duration of psychotherapy was shorter than in a prior study showing benefits (mean 12.5 vs. 16) [226,227]. In the STAR*D study, the remission rate for cognitive therapy (either in combination with or as monotherapy) following initial treatment with citalopram was as effective as pharmacologic augmentation [229], though it was underpowered to detect between-group differences; only a quarter of patients completed the full 16 session protocol, and only 26% of participants agreed to assignment in cognitive therapy randomization groups. Given the tolerability of psychotherapy and the potential benefit, combined treatment should be considered in all patients with TRD [225,226,227,228].

Recommendation: concomitant psychotherapeutic intervention is strongly encouraged.

## 9. Conclusions and Recommendations for Practicing Personalized Medicine

### 9.1. General 

In the initial assessment of suspected TRD, a comprehensive diagnostic assessment should be conducted to include physical examination and medical history with an emphasis on duration, dosing and adherence to prior treatment trials. Social and family histories should be carefully reviewed, with the inclusion of life events, occupational history, environmental stressors and personality factors, as well as collateral sources of information from family members, medical records and prior clinicians included where appropriate, as patients may not be capable of providing sufficiently detailed information. Medical history should include the standard components: medical problems; prior hospitalizations; prior surgeries; current medications; toxic exposures; and prior injuries, particularly head injuries or loss of consciousness, in order to appropriately identify underlying medical conditions or iatrogenic causes of depression, such as hypothyroidism. Laboratory testing should be conducted and tailored to the patient’s history, and may include a complete blood count, iron studies, serum electrolytes, liver function tests, blood urea nitrogen, creatinine, thyroid function tests, serum B12, folate levels and toxicology studies as appropriate. For some patients, more extensive endocrine and infectious disease testing or polysomnography may be indicated. Validation of diagnosis through the assessment of symptomatology, heredity, course of illness and treatment response is crucial for differential diagnosis to identify co-morbidities and to rule out BD that may masquerade as treatment-resistant MDD. 

### 9.2. Specific

**Figure 4:**
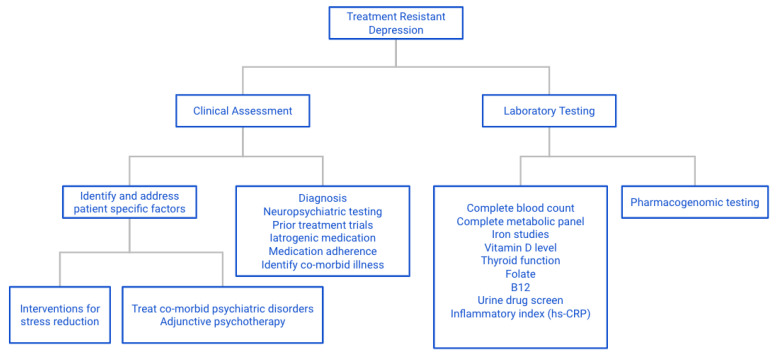
Flow chart for assessment of TRD: approach to assessing possible contributing factors to TRD.

Based on the preceding discussion and review of pertinent publications, we recommend the following specific tests that can be obtained in most medical centers, either on site or through commercial laboratories (Figure 4). They include the measurement of blood levels of specific vitamins, notably, B9, B12 and D. Pharmacogenomics testing is strongly recommended, as increasingly, such tests are covered by health insurance plans with generally affordable co-pays. The continued use of substances known to interact with pharmacogenomic and pharmacokinetic parameters must be strongly discouraged, and where indicated, referral of the patient to rehabilitation services to achieve permanent detoxification and abstinence should be considered. 

## Data Availability

Not applicable.
